# Decreased modulation by the risk level on the brain activation during decision making in adolescents with internet gaming disorder

**DOI:** 10.3389/fnbeh.2015.00296

**Published:** 2015-11-03

**Authors:** Xin Qi, Xin Du, Yongxin Yang, Guijin Du, Peihong Gao, Yang Zhang, Wen Qin, Xiaodong Li, Quan Zhang

**Affiliations:** ^1^Department of Radiology and Tianjin Key Laboratory of Functional Imaging, Tianjin Medical University General Hospital Tianjin, China; ^2^Department of Psychology, Linyi Fourth People’s Hospital Linyi, China; ^3^Department of Radiology, Linyi People’s Hospital Linyi, China

**Keywords:** internet gaming disorder, BART, dorsal lateral prefrontal cortex, fMRI, risky decision-making

## Abstract

Greater impulse and risk-taking and reduced decision-making ability were reported as the main behavioral impairments in individuals with internet gaming disorder (IGD), which has become a serious mental health issue worldwide. However, it is not clear to date how the risk level modulates brain activity during the decision-making process in IGD individuals. In this study, 23 adolescents with IGD and 24 healthy controls (HCs) without IGD were recruited, and the balloon analog risk task (BART) was used in a functional magnetic resonance imaging experiment to evaluate the modulation of the risk level (the probability of balloon explosion) on brain activity during risky decision making in IGD adolescents. Reduced modulation of the risk level on the activation of the right dorsolateral prefrontal cortex (DLPFC) during the active BART was found in IGD group compared to the HCs. In the IGD group, there was a significant negative correlation between the risk-related DLPFC activation during the active BART and the Barratt impulsivity scale (BIS-11) scores, which were significantly higher in IGD group compared with the HCs. Our study demonstrated that, as a critical decision-making-related brain region, the right DLPFC is less sensitive to risk in IGD adolescents compared with the HCs, which may contribute to the higher impulsivity level in IGD adolescents.

## Introduction

Internet gaming disorder has become increasingly prevalent around the world, especially in Asia (Wu et al., 2013; Tang et al., 2014), and results in an adverse impact on various behavioral and psychosocial aspects (Ko et al., 2010). Behavioral research suggested that a reduced risky decision-making ability is one of the most important behavioral impairments in IGD individuals (Pawlikowski and Brand, 2011; Yao et al., 2015). For instance, researchers found that IGD individuals made more disadvantageous choices on the Game of Dice Task compare with HCs and that such an impairments may be partly the result of failure to utilize feedback (Pawlikowski and Brand, 2011; Yao et al., 2014). Moreover, studies revealed that IGD subjects show a diminished consideration of experiential outcomes when making future decisions (Dong et al., 2013). Risky decision making is a high-level cognitive function and is essential for human survival in an uncertain environment (Hastie, 2001). Risk aversion is an essential part of the decision-making process in a normal population (Macoveanu et al., 2013). However, IGD individuals tend to exhibit disadvantageous risky decision making and encounter more adverse situations (Yao et al., 2015), which may lead to a negative effect on IGD individuals and society. Therefore, it is important to investigate the neural mechanisms underlying the altered risky decision making in IGD individuals.

The neural circuits related to risky decision making were wildly examined in healthy subjects, and a distributed subcortical-cortical network mainly consisting of prefrontal, parietal, limbic, and subcortical regions was found to be involved in risky decision making (Ernst and Paulus, 2005; Trepel et al., 2005; Krain et al., 2006; Rao et al., 2008; Kohno et al., 2015), and the brain activation levels in these regions were found to be associated with the risk level (Schonberg et al., 2012; Galván et al., 2013; Telzer et al., 2013a; Rao et al., 2014; Kohno et al., 2015). However, few neuroimaging studies focused on the effect of IGD on the neural substrates for risky decision making. A fMRI study by Dong et al. (2013) found that individuals with internet addiction disorder required more brain resources to complete the decision-making task and ignored the feedback of previous outcome, which is an essential feature of risky decision making in HCs. A study by Lin et al. (2015) revealed that activation levels of the left inferior frontal gyrus and left precentral gyrus decreased in IGD individuals when performing a probability discounting task, which suggested impaired risk evaluation in IGD individuals. Although these studies suggested that the IGD is associated with abnormal brain activity during risky decision-making processes, how the risk level modulates brain activation during decision making is still poorly understood in IGD individuals. To our knowledge, no study thus far focused on the covariance between brain activation and the risk levels during the decision-making process in IGD individuals, which may advance current understanding of the mechanisms underlying decision-making deficits in IGD individuals.

In this study, 23 IGD adolescents and 24 HCs were enrolled, and fMRI data were obtained while the participants performed the BART (Lejuez et al., 2002) to evaluate how the risk level modulates brain activation during decision-making processes in IGD adolescents compared to the HCs. The BART, in which participants inflate virtual balloon that can either grow larger or explode, provides an ecologically valid model to assess human risk-taking propensity and behavior and provides participants with a choice in determining the risk level for each balloon; the larger the balloon was inflated, the greater the risk that the participants are taking. Unlike other risk tasks, the risk in the BART was more directly and ecologically defined as the probability of explosion for each balloon; thus, the BART is adaptive in terms of evaluating the modulation of the risk level on brain activation during the decision-making process. The BART has been successfully used in healthy volunteers, and multiple brain regions were demonstrated to be related to the risk, including the DLPFC, ventromedial prefrontal cortex, ACC/medial frontal cortex, striatum, and insula (Rao et al., 2008; Schonberg et al., 2012; Helfinstein et al., 2014; Kohno et al., 2015). The BART has also been used in addiction studies, and the abnormal brain activation was detected in the DLPFC and striatum of methamphetamine-addicted individuals (Kohno et al., 2014), and in the prefrontal cortex and ACC of alcohol-dependence individuals (Bogg et al., 2012; Claus and Hutchison, 2012). As a special behavioral addiction (Karim and Chaudhri, 2012; Carli et al., 2013), IGD may also affect the activity in the risk-related brain regions. Thus, in this study, we used fMRI with BART to investigate whether the modulation of the risk level on the brain activation during the decision-making process is altered in IGD adolescents when compared to HCs. This study will contribute to the understanding of the neuro mechanisms of the risk-taking and impulsive behaviors in IGD adolescents.

## Materials and Methods

### Participant Selection

Because the diagnostic standards for IGD are still ambiguous (Blaszczynski, 2008; Griffiths, 2008), relatively strict inclusion criteria were selected in this study. First, the YDQ for internet addiction (Young, 1998) was used to determine the presence of an internet addiction disorder. YDQ consisted of eight “yes” or “no” questions regarding internet use. Participants who reported five or more “yes” answers were diagnosed as having an internet addiction disorder (Young, 1998). A score of 50 or higher on IAT (Young and Internet Addiction Test [IAT], 2009) was used as the second inclusion criteria. In addition, only IGD adolescents who reported themselves as spending an average of four or more hours/day playing internet games (>80% of total online time) were recruited. According to these inclusion criteria, 26 right-handed male IGD adolescents were recruited in this study. Only the male subjects were examined because of the relatively small number of females with internet gaming experience. Twenty-five male participants were recruited as HCs. HCs were defined as subjects who did not fit the criteria for a YDQ diagnosis, spend less than 2 h per day on the internet, and whose IAT score was less than 50. All the participants were medication free, and reported no history of substance abuse or head injuries. The impulsivity was evaluated for all participants with the BIS-11 (Patton et al., 1995). The IQ of all participants was tested using SPM. The data from three of 26 IGD adolescents and one of 25 HCs were discarded from this study because of obvious head motion during the fMRI experiment (maximum displacement in any cardinal directions is more than 2 mm and/or maximum spin is more than 2°). The data for the remaining 23 IGD adolescents and 24 HCs were used for further analysis. Age, education, and IQ were matched well between the two groups, and the BIS scores and IAT scores were significantly higher in IGD group than in HCs (**Table 1**).

**Table 1:** Demographic and clinical characteristics of subjects (Mean ± SD).

	IGD (*N* = 23)	HCs (*N* = 24)	*t*	*P*
Age (year)	17.26 ± 3.56	17.42 ± 3.05	-1.161	0.872
Education (year)	10.13 ± 3.04	11.25 ± 2.88	-1.298	0.201
IQ (SPM)	48.78 ± 6.91	48.58 ± 6.26	0.104	0.918
IAT score	70.35 ± 10.76	33.42 ± 7.75	13.545	<0.001
BIS total	68.17 ± 11.70	54.13 ± 8.05	4.816	<0.001

*Two-sample two-tailed t-test. Significant level is set as P < 0.05*.

*BIS, Barratt impulsivity scale; HCs, healthy controls; IAT, internet addiction test; IGD, internet game disorder; IQ, intelligence quotient; SPM, standard Raven’s progressive matrices*.

This study was approved by the Ethical Committee of Tianjin Medical University General Hospital and written informed consent was obtained from all participants or their guardians.

### Task and Procedure

In the present study, we adapted the fMRI-adapted version of the BART used by Rao et al. (2008). Briefly, the participants were presented a virtual balloon and asked to press one of two buttons to either inflate (pump) the balloon or cash out. The larger balloons were associated with greater rewards and greater risk of explosion. Participants could stop inflating the balloon at any point to win the wager or continue inflation until the balloon explodes, in which case they lose the wager. The maximum number of pumps that participants could use for each balloon was 12. A control cue (the color of a small circle changed from red to green) was used to instruct the participants to begin inflation. After the participants successfully pressed a button and pumped the balloon, the small circle immediately turned red at a random interval of between 1.5 and 2.5 s. The cue then turned green again to indicate the next inflation period. After the end of each balloon trial, there was also a varying 2–4 s interval before the next balloon trial. The win or loss picture was presented for 1.5 s. The picture of the exploded balloon was presented for 20 ms. The risk of balloon explosion (the probability of balloon explosion) was defined as the “risk level.” The covariance between the risk level and the activation of the brain regions was defined as the “modulation.”

We used two modes of BART in our study: active choice and passive no-choice modes. In the active choice mode, the participants could determine the risk level and decided to either inflate the balloon or cash out. However, in the passive no-choice mode, the participants merely inflated the balloon continually while the computer determined the end point as well as the win or loss for each balloon. The number of balloons that participants completed during the scan was not pre-determined but depended on the response speed in either active or passive modes. The only difference between the two modes is the option in the active mode to discontinue the inflation and win the wager. The brain activation levels of the active choice mode compared to the passive no-choice mode (active-passive) reflect the neural basis of the decision-making process. After the experiment, the participants received the equivalent amount of money earned during the active mode experiment.

### Data Acquisition

The functional MRI was conducted on a Siemens 3.0T scanner (Magnetom Verio, Siemens, Erlangen, Germany) using a gradient-recalled echo planar imaging sequence with the following parameters: repetition time (TR) = 2000 ms, echo time (TE) = 30 ms, field of view = 220 mm × 220 mm, matrix = 64 × 64, slice thickness = 4 mm, and slice gap = 1 mm. The task stimuli were projected onto a display screen in front of the magnet’s bore and the participants viewed the stimuli through a mirror installed on the head coil. The participants responded to the task by pressing the button on the fMRI-compatible response box. The formal experiment was performed after the participants learned and practiced the tasks. All of the participants completed two 10 min functional runs, one for each task mode. The scanning order of the two tasks was counterbalanced across the participants within each group.

### Behavioral Analysis

In the fMRI experiment, the behavioral variables of the BART included the trial number, total and mean number of pumps, number of wins and losses, adjusted number of pumps (defined as the average number of pumps excluding the balloons that exploded), the reward collection rate (the number of win trials divided by the number of total trials), and average RT for all pumps. Only behavioral data during the active mode was analyzed because the participants were forced to accept the outcome determined by computer for each balloon during the passive mode. A two-sample *t*-test was used to compare the difference in the behavioral data during the active mode between the IGD individuals and the HCs. Statistical analyses were conducted with SPSS 21.0, and the significance level was set at *P* < 0.05.

### Functional MRI Data Preprocessing

Functional MRI data preprocessing were conducted using SPM8 (http://www.fil.ion.ucl.ac.uk/spm/software/spm8). For each participant, the functional images were corrected for the acquisition time delay between the different slices and corrected geometric displacements according to the estimated head movement. The images were then realigned with the first volume. Based on the motion correction estimates, participants who demonstrated a maximum displacement in any of the x, y, or z directions greater than 2-mm or more than 2° of angular rotation (x, y, or z) were excluded from this study. Following this step, all realigned images were spatially normalized to the MNI EPI template, resampled to 3 mm × 3 mm × 3 mm, and subsequently smoothed with a 6 mm FWHM.

### Statistical Analysis

The GLM was used for voxel-based individual data analysis. The BOLD time series data were modeled using a standard HRF with a time derivative. The head movement parameters of each subject were modeled as covariates of no interest. A high-pass filter with a cut-off at 128 s was used to remove low frequency fluctuations.

The GLM included three types of events resulted from a button press: an inflation of the balloon, a win outcome, or a loss outcome. Thus, the GLM for either active or passive task included three regressors that represent three types of events, respectively. The risk level associated with each inflation (i.e., the probability of explosion, orthogonalized by a mean central correction) was also entered into the model as a linear parametric modulation of the balloon inflation regressor. For each subject, the risk-related contrast in active and passive tasks was defined to examine the brain activations that covaried with the risk level.

The second level random effect analyses were conducted by using a 2 (group: IGD and HCs) × 2 (choice mode: active and passive) ANOVA on the risk-related contrasts with full factorial in SPM8, and the risk-related contrasts in the active and passive modes within the same participant were processed as repeated measures. In this study, the main aim was to evaluate the intergroup difference of the risk-related brain activation during the decision-making process, which can be reflected by the activation seen in the active mode compared to the passive mode (active–passive). Therefore, the interactive effect between the group and choice mode, HCs (active–passive) – IGD (active–passive), was analyzed in this study. A correction for multiple comparisons was performed using the Monte Carlo simulation, resulting in a corrected threshold of *P* < 0.05 (AlphaSim program, parameters including: single voxel *P* = 0.005, 1000 simulations, full width at half maximum = 6 mm, cluster connection radius *r* = 5 mm, and the mask of global gray matter). The brain regions with interactive effects were established as ROIs. The average β estimates within ROIs were extracted and a *post hoc t*-test was conducted.

The correlation between the average β estimates within ROIs, BIS scores, and IAT scores were examined with a Pearson’s correlation analysis in IGD group with SPSS 21.0. The significance level was set at *P* < 0.05.

## Results

### Behavioral Results

**Table 2** shows the behavioral results during the fMRI experiment. The two-sample *t*-test revealed that the average RT was shorter in IGD group than in the HCs while the active mode took place (*P* = 0.03), the number of the total pumps was significantly more in the IGD group (*P* < 0.001). There was no significant difference in the adjusted number of pumps, trial number, mean number of pumps, number of wins and losses, and the reward collection rate.

**Table 2:** The behavioral results of the BART during active functional magnetic resonance imaging (fMRI) experiment (Mean ± SD).

	IGD (*N* = 23)	HCs (*N* = 24)	*t*	*P*
Mean pumps	7.74 ± 1.01	7.32 ± 0.93	1.471	0.147
Adjusted pumps	6.23 ± 1.12	5.71 ± 1.10	1.614	0.114
Total pumps	211.00 ± 9.84	199.21 ± 15.16	3.147	<0.001
Trial number	31.39 ± 5.03	31.58 ± 5.03	-0.131	0.897
Win trials	23.52 ± 5.90	25.00 ± 7.02	-0.780	0.440
Pop trials	7.87 ± 3.18	6.58 ± 2.81	1.470	0.148
Reward collection rate	0.75 ± 0.11	0.78 ± 0.12	-1.036	0.306
Response time (ms)	492.38 ± 72.83	553.21 ± 110.23	-2.222	0.031

*Significant level is set as P < 0.05*.

### Imaging Results

**FIGURE 1:**
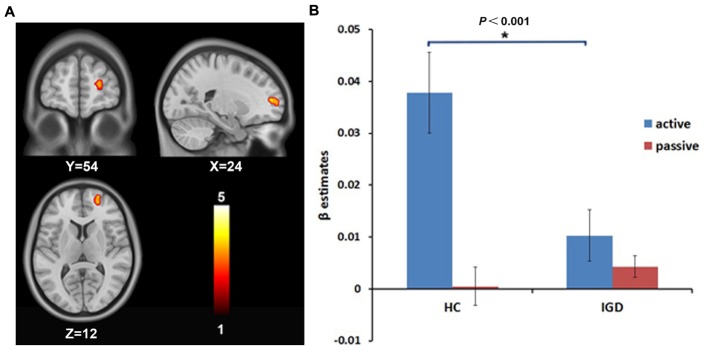
**The intergroup difference in the modulation by the risk level on the brain activation of the right dorsolateral prefrontal cortex (DLPFC). (A)** The modulation by the risk level on the brain activation of the right DLPFC shows intergroup difference. **(B)** Region of interest (ROI) analysis shows that the modulation by the risk level on the activation of the right DLPFC decreased during the active decision-making process in internet gaming disorder (IGD) group compared to HCs.

**FIGURE 2:**
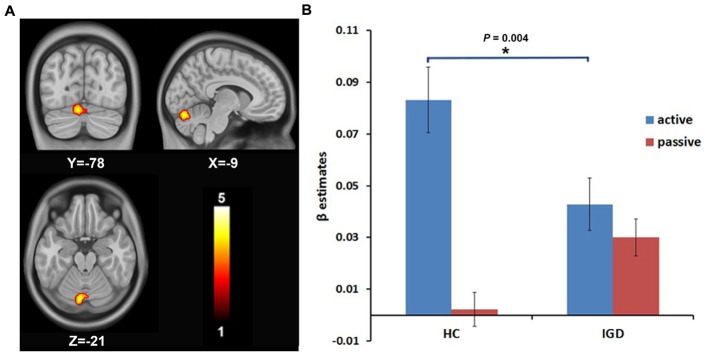
**The intergroup difference in the modulation by the risk level on the brain activation of the left cerebellum. (A)** The modulation by the risk level on the brain activation of the left cerebellum shows intergroup difference. **(B)** ROI analysis shows that the modulation by the risk level on the activation of the left cerebellum decreased during the active decision-making process in IGD group compared to HCs.

A 2 (group: IGD and HCs) × 2 (choice mode: active and passive) ANOVA on the risk-related contrasts revealed a significant interactive effect on the activation of the right DLPFC (MNI coordinate: 24, 54, 12; voxels: 38; *t* = 3.78; *P* < 0.05, AlphaSim correction; **Figure 1A**). The *post hoc t*-test revealed that the modulation of the risk level on the activation of the right DLPFC was higher in active mode than in passive mode in HCs, but showed no significant difference between the active and passive modes in the IGD group. During the active mode, the modulation of the risk level on the activation of the right DLPFC significantly decreased in the IGD group compared with the HCs (**Figure 1B**). In addition, a significant interactive effect was also found for the activation of the left cerebellum (MNI coordinate: -9, -78, -21; voxels: 72; *t* = 4.13; *P* < 0.05, AlphaSim correction; **Figure 2A**). The *post hoc t*-test revealed that the difference in the modulation of the risk level on the activation of the left cerebellum between the modes and between the groups had similar features to whose seen in the right DLPFC (**Figure 2B**).

**FIGURE 3:**
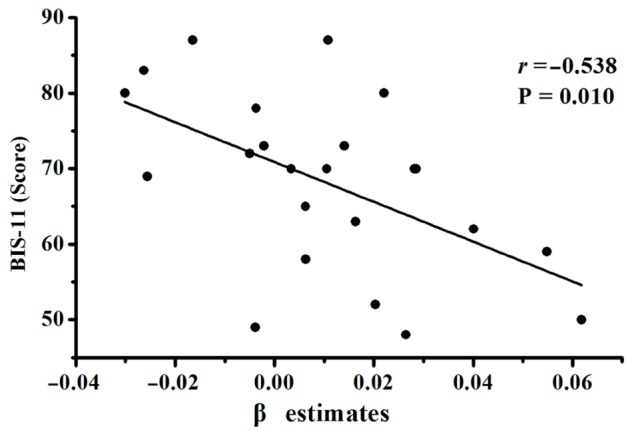
**Correlation between the β estimates within the ROI of the right DLPFC and Barratt impulsivity scale (BIS) total scores in IGD group**.

The modulation of the risk level on the activation of the right DLPFC during the active mode showed a significantly negative correlation with the BIS total scores in the IGD group (**Figure 3**). There was no significant correlation between the activation of the right DLPFC and IAT scores in the IGD group. In addition, no significant correlation was found between the fMRI results and the behavioral data during decision making.

## Discussion

To our knowledge, this is the first study to evaluate the modulation of the risk level on the brain activation during the decision-making process in IGD adolescents by using a BART fMRI. Decreased risk-related activations of the right DLPFC during active decision making were found in the IGD group compared with the HCs, which suggested that the activation of the right DLPFC was less sensitive to the risk level in the IGD group than in the HCs. The modulation of the risk on the activation of the right DLPFC during the active decision-making process was negatively correlated with the BIS score in the IGD group. These findings may contribute to the understanding of the neural mechanisms of higher impulsivity in IGD adolescents.

Risky decision making likely draws upon several brain processes that are involved in the estimations of value and risk, executive control, and emotional processing (Gowin et al., 2013). The DLPFC is a critical brain region involved in executive control (Bari and Robbins, 2013; Yuan and Raz, 2014) that regulates goal-oriented, flexible, and effective behavior and may mediate decision making with explicit risk (Brand et al., 2006; Schiebener et al., 2012). The altered structure and function of the DLPFC has been demonstrated in IGD individuals (Yuan et al., 2011; Ko et al., 2013; Liu et al., 2014), which were consistent with the findings in studies on substance addiction (Bolla et al., 2005; Moeller et al., 2014) and behavioral addiction (Crockford et al., 2005). During decision making, the DLPFC activity may mediate the integration of information about risk and value (Gowin et al., 2013), represent prospects, evaluate outcomes, and calculate the subsequent utility (Trepel et al., 2005). The IGD adolescents usually presented with impaired executive control ability (Zhou et al., 2012; Dong et al., 2015); therefore, it is plausible to postulate that the decreased risk-related activation of the right DLPFC during risky decision making in IGD adolescents may reflect the impaired executive control function that mediated adverse choices during risky situations. In this study, the right but not the left DLPFC showed decreased risk-related activation in IGD adolescents compared with the HCs. This laterality of the right as opposed to the left DLPFC activity mediating risky decision making was also reported in other BART fMRI studies (Rao et al., 2008; Schonberg et al., 2012; Galván et al., 2013; Kohno et al., 2014) and the transcranial direct-current stimulation studies (Gorini et al., 2014). Furthermore, this laterality of decreased activation in the right DLPFC was also found in drugs-addicted individuals when they performed a series of risky decision-making tasks (Bolla et al., 2005; Ersche et al., 2005; Kohno et al., 2014). Taken together, these results implicated that the right DLPFC was a key region for risky decision making, and the possible neural mechanism underlying the alteration of the DLPFC activation in IGD adolescents may be similar to that in individuals with substance abuse issue.

Recently, the IGD has been conceptualized as a behavioral addiction or an impulse-control disorder (Kuss, 2013; Ding et al., 2014), and may be associated with inhibition function impairment (Dong and Potenza, 2014; Liu et al., 2014), which is similar to that in the other behavioral addiction (Grant et al., 2010), such as pathological gambling (Miedl et al., 2012; Kräplin et al., 2014). A review suggested that impulsive inhibition is a part of decision-making function (Sakagami et al., 2006), and research has successfully demonstrated that the DLPFC has an important role in the impulsive inhibition process (Asahi et al., 2004; Garavan et al., 2006; Nakata et al., 2008a,b; Probst and van Eimeren, 2013). In the current study, the higher BIS-11 scores in IGD individuals compared with the HCs implicated a higher impulsivity in IGD adolescents, which was consistent with the findings in other studies on impulsive control in IGD individuals (Ding et al., 2014; Ko et al., 2014; Metcalf and Pammer, 2014). Therefore, the decreased modulation of the risk level on the activation of the right DLPFC in IGD adolescents in our study may be associated with impulsive inhibition impairments. Furthermore, a significant negative correlation was found between the decreased modulation of the risk level on the activation of the right DLPFC during the active choice and the BIS-11 score in IGD adolescents, which means that the IGD adolescents with higher impulsivity showed lower modulation of the risk level on activation of the right DLPFC during the decision-making process. The right DLPFC activation was less sensitive to risk during the decision-making process in IGD adolescents with higher impulsive propensities. The decreased modulation of the risk level on the activation of the right DLPFC in IGD adolescents may mediate their ignoring the risk.

Our study found that, besides the right DLPFC, the modulation of the risk level on the activation of the left cerebellum also decreased during the active decision-making process in the IGD group. Although alterations in cerebellum activation had been reported in previous fMRI studies with BART (Galván et al., 2013; Telzer et al., 2013a,b; Rao et al., 2014) and other tasks that involved the decision-making processes (Rosenbloom et al., 2012; Gabay et al., 2014), the neural mechanism has not been clearly determined. Previous studies have found that the cerebellum is a critical component in addiction issues (Kühn et al., 2012; Moulton et al., 2014), and the gray matter volume of the cerebellum, especially left cerebellum, reduced in subjects with substance disorder (Moreno-López et al., 2015). Moreover, the decreased gray matter volume (Wang et al., 2015) and the enhanced regional homogeneity (Dong et al., 2012) in the left cerebellum has also been reported in IGD individuals. Therefore, it is worth performing further studies involved in the association between the cerebellum activity and the risky decision making in IGD individuals.

Several limitations should be considered in the present study. First, the sample size was relatively small, which may reduce the power and fail to detect some brain activations with slight significance. Second, the maximum number of possible balloon pumps in this modified BART task was reduced to 12, and most participants completed only about 30 balloon trials during the 10 min of BOLD scanning. Thus, the limitations inherent in this experimental design may have decreased the sensitivity of detecting intergroup differences in behavioral performance (Rao et al., 2010). Finally, the causal relationship between the altered brain activation and IGD cannot be determined with this cross-sectional study. A longitudinal study may be helpful for evaluating this relationship.

## Conclusion

This is believed to be the first study to test the modulation of the risk level on brain activation during the decision-making process with the BART in IGD adolescents. Our study demonstrated that the modulation of the risk level on the activation of the right DLPFC decreased in IGD adolescents, and the decreased risk-related activation of the right DLPFC was negatively correlated with the BIS scores. Our findings suggested that, as a critical brain region related to the decision making, the right DLPFC is less sensitive to the risk level in IGD adolescents compared with the HCs, which may contribute to the higher impulsivity in IGD adolescents.

## Author Contributions

XQ, YY, XL, and QZ designed research; XQ, XD, PG, YZ, GD, and QZ performed research; YY, PG was involved in the clinical assessment; XQ, YZ, GD, WQ, and QZ analyzed data; XQ, YZ, XL, YY, and QZ wrote the paper.

## Conflict of Interest Statement

The authors declare that the research was conducted in the absence of any commercial or financial relationships that could be construed as a potential conflict of interest.

## ABBREVIATIONS

ACC: anterior cingulate
BART: balloon analog risk task
BIS-11: Barratt impulsivity scale
DLPFC: dorsolateral prefrontal cortex
fMRI: functional magnetic resonance imaging
FWHM: full-width at half-maximum
GLM: general linear model
HC: healthy controls
HRF: hemodynamic response function
IAT: Young’s online internet addiction test
IGD: internet gaming disorder
IQ: Intelligence Quotient
MNI: Montreal Neurological Institute
ROI: region of interest
RT: response time
SPM: Standard Raven’s ProgressiveMatrices
SPM8: Statistical Parametric Mapping software
YDQ: Young Diagnostic Questionnaire
