# Corrigendum: Decreased modulation by the risk level on the brain activation during decision making in adolescents with internet gaming disorder

**DOI:** 10.3389/fnbeh.2019.00074

**Published:** 2019-04-09

**Authors:** Xin Qi, Xin Du, Yongxin Yang, Guijin Du, Peihong Gao, Yang Zhang, Wen Qin, Xiaodong Li, Quan Zhang

**Affiliations:** ^1^Department of Radiology and Tianjin Key Laboratory of Functional Imaging, Tianjin Medical University General Hospital, Tianjin, China; ^2^Department of Psychology, Linyi Fourth People's Hospital, Linyi, China; ^3^Department of Radiology, Linyi People's Hospital, Linyi, China

**Keywords:** internet gaming disorder, BART, dorsal lateral prefrontal cortex, fMRI, risky decision-making

In the original article, there was an error. The statement of written informed consent procedures was incorrect because of inappropriate wording.

A correction has been made to the **Materials and Methods**, subsection **Participant Selection**, paragraph two:

“This study was approved by the Ethical Committee of Tianjin Medical University General Hospital and written informed consent was obtained from all participants or their guardians.”

The authors apologize for this error and state that this does not change the scientific conclusions of the article in any way. The original article has been updated.

